# Cramp–Fasciculation Syndrome Associated with Natural and Added Chemicals in Popular Food Items

**DOI:** 10.3390/foods13142257

**Published:** 2024-07-17

**Authors:** Emmeline Lagrange, Jean-Paul Vernoux, Celia Chambon, William Camu, Peter S. Spencer

**Affiliations:** 1 Department of Neurology, Reference Center of Neuromuscular Disease and ALS Consultations, Grenoble University Hospital, 38043 Grenoble, France; cchambon@chu-grenoble.fr; 2 Unité de Recherche Aliments Bioprocédés Toxicologie Environnements (ABTE) EA 4651, Normandie University, UNICAEN, 14000 Caen, France; 3 The Neuroscience Institute of Montpellier, Inserm UMR1051, 34000 Montpellier, France; 4 Department of Neurology, School of Medicine, Oregon Health & Science University, Portland, OR 97201, USA; spencer@ohsu.edu

**Keywords:** muscle cramp–fasciculations, motoneuron disease, glutamate, lupin

## Abstract

Cramp–fasciculation syndrome (CFS) is a rare and benign neuromuscular disorder that may initially masquerade as motor neuron disease/amyotrophic lateral sclerosis. While CFS may have a familial disposition, we report on cases associated with high consumption of popular food items. One set of patients reversibly experienced acute onset of headache, flushing, muscle stiffness and fasciculations following the consumption of umami-flavored food containing a large concentration of monosodium glutamate. A second group of patients consuming food derived from lupin seed developed acute cholinergic toxicity, CFS, and, with chronic intake, significant, self-limiting, but incompletely reversible upper and lower motor neuron deficits. While these cases may improve our knowledge about the possible causes of CFS, our series also demonstrates that excessive consumption of some popular foods is not harmless. This warrants further research on their safety at all stages of human development from a neurological point of view.

## 1. Introduction

Cramp–fasciculation syndrome (CFS) is an essential/benign and generally self-limiting disorder characterized by muscle fasciculation and cramping without the development of muscle weakness or atrophy [1]. The syndrome is rare, affecting 0.4–0.7% in a healthy population with a sex ratio in disfavor of women [2,3]. Fasciculations are discreet, rapid, repetitive, non-painful, and localized limb muscle contractions that often occur in isolation or may be associated with muscle cramping. Skeletal muscle cramps are sudden, involuntary, painful, and palpable muscle contractions lasting seconds to minutes that are relieved by voluntary limb extension. CFS is considered to represent a hyperexcitable peripheral nerve syndrome, although the neurogenic origins of the underlying motor discharges may vary [4]. Muscle cramps, fasciculation, and myokymia result from peripheral nerve hyperexcitability in Isaacs syndrome, some cases of which have serum antibodies against voltage-gated potassium channels [5]. An apparently autosomal dominant muscle syndrome without fasciculations, thought to result from a large-fiber peripheral neuropathy, has been described in a Japanese family [6]. Muscle twitching and spasms occur in hypothyroid myopathy [7], and pregnancy can unmask a variety of sub-clinical neuromuscular disorders, including amyotrophic lateral sclerosis (ALS) [8]. Muscle fasciculation is so closely associated with this rare, progressive, and fatal neuromuscular disease that the de novo appearance of muscle fasciculation may generate an anxiety state known as Fasciculation Anxiety Syndrome in Clinicians [9,10].

Consistent with the proposal that CFS results from peripheral nerve hyperexcitability, we report cases of transitory food-related CFS linked to excessive ingestion of either monosodium glutamate (MSG) or the seed of white lupin (*Lupinus* spp.) These cases illustrate the health hazards of certain popular food practices and mandate research on their safety for brain health at all stages of human development.

## 2. Case Report

A total of six cases is reported in this study; they were observed in 2016 and 2021 in the Grenoble town area in France. They were between 36 and 56 years old, and five out of six of the cases were women. They all consulted the department of neurology at the Grenoble University Hospital in France for cramp–fasciculation syndrome and were received by Dr Lagrange and Dr Chambon. Cases were described based on a questionnaire related to their food and corresponding intake amount, the time interval between intake and symptoms, the duration of the symptoms, the description of the cramp–fasciculation and associated symptoms, and previous susceptibility (Table 1).

These cases were analyzed and compared to previously reported cases of cramp–fasciculation associated with food ingestion.

## 3. Results and Discussion

### 3.1. Description of Cases Involving Glutamate as a Dietary Excitotoxin

In 2021, we received three health professional colleagues with acute CFS that began within a few hours of sharing a meal in a Chinese restaurant. The disorder was painful and featured nocturnal leg cramps and tightness; cramps and fasciculations were described as day-long and lasting for 3 days, interfering with daily activities and sleep. Vertigo, suffocation, chest-oppression migraine, hypersalivation, and hot flashes were variably experienced the first night after their meal. The subjects were thought to have a food allergy and therefore treated with anti-histamines, which proved to be therapeutically ineffective. Recovery was spontaneously completed by day 4 when the patients each had a normal neurological examination, including electromyography (EMG) of six muscles. 

The Chinese restaurant reported that the three colleagues had consumed a shared meal that contained a massive quantity of umami (half a pound), a flavor enhancer containing monosodium glutamate (MSG). Among the three colleagues, two had already eaten Chinese food without adverse effects, but the third had previously experienced an exacerbation of her migraine after consuming soya sauce, which contains a high level of MSG. Furthermore, there was some regular contact with MSG in their weekly food intake resulting from the ingestion of food items with a high natural content of MSG, such as tomatoes, broccoli, or Roquefort cheese. 

The three subjects were diagnosed with MSG syndrome complex given the evidence of excess umami consumption, a temporally related onset of a mostly common cohort of symptoms (headache, flushing, muscle tightness, and fasciculation), their spontaneous disappearance, and, for one subject, a prior sensitivity to MSG. 

The symptoms presented by our three cases add to the original descriptions of Chinese Restaurant Syndrome, attributed to the responses of only sensitive individuals to high MSG intake [11] which emphasized the acute onset of burning sensations, facial pressure, and chest pain [12]. While toxic effects resulting from the ingestion of a large dose of MSG are recognized, its use as a food additive is considered by food safety regulatory agencies to be a safe practice. The European Food Safety Organization (EFSA) [13] regularly (every ten years) reassesses the safety of glutamic acid and glutamates in food: in 2017, an acceptable daily intake (ADI) of 30 mg/kg bw/day expressed as glutamic acid was established. The EFSA’s expert scientific panel noted that “the exposure to glutamic acid (E620) and glutamates (E621–E625) [sodium glutamate/MSG (E621), potassium glutamate (E622), calcium glutamate (E623), ammonium glutamate (E624) and magnesium glutamate (E625)] exceeded not only the proposed ADI, but also doses associated with adverse effects in humans for some population groups”. These include “the MSG symptom complex (>42.9 mg/kg bw per day), headache (85.8 mg/kg bw per day), increased blood pressure (150 mg/kg bw per day) and increased insulin (>143 mg/kg bw per day)”. Importantly, the ingestion of E620–625 compounds may be continuous, since the compounds are widely used as flavor enhancers not only in commercial food products (bakery wares, soups and broths, sauces, meat and meat products, seasoning and condiments, and food supplements) but also in nutriments such as tomatoes, cheeses, and grapes. MSG is also present in soya sauce, in autolyzed yeast extracts, and in proteins hydrolyzed with glutaminase, an enzyme that converts glutamine to glutamic acid. Glutaminase is a regulated food additive in Canada and a processing aid in Europe [14] for products such as wheat flour, bakery products, pasta, hydrolyzed proteins, egg products, and aromatic preparations. E620–E625 are forbidden in infant foods, a decision based on the application of the Precautionary Principle. 

Glutamate is the major excitatory neurotransmitter throughout the human and mammalian nervous system. The neurotransmitter glutamate interacts with ionotropic (NMDA, AMPA, kainate) and metabotropic receptors on post-synaptic neurons [15]. The concentration of synaptic and extra-synaptic glutamate is low and tightly controlled since excess amounts of the excitatory amino acid induces the hyperexcitability of post-synaptic neurons that may result in cell death, a phenomenon known as excitotoxicity [16,17]. Human neurological diseases associated with excitotoxicity include (i) lathyrism, a self-limiting upper neuron disorder triggered by chronic oral exposure to the AMPA-receptor agonist β-*N*-oxalylamino-L-alanine (BOAA) in the food plant grasspea (*Lathyrus sativus*) [18] and (ii) amnesic shellfish poisoning featuring seizures and permanent loss of short-term memory caused by oral exposure to domoic acid, an AMPA- and kainite-receptor agonist produced by *Pseudo-nitzschia* sp., a marine diatom that populates shellfish [19]. There is also a potential mechanistic link between glutamate excitotoxicity and ALS, a fatal motor system disease characterized by fasciculation, progressive weakness, and atrophy of musculature that is treated with 2-amino-6-trifluoromethoxy benzothiazole (Riluzole), a drug that inhibits the pre-synaptic release of the glutamate neurotransmitter [20]. Preclinical studies investigating oral glutamate neurotoxicity in mice showed not only neuronal damage in the cerebrum, hippocampus, and cerebellum, but also decreased brain levels of superoxide dismutase (SOD) [20]. SOD is a key antioxidant enzyme protecting cells from the deleterious effects of superoxide radicals, especially in the mitochondria. SOD mutations are responsible for some familial ALS cases, both through a detrimental loss of activity of the enzyme leading to glutamate excitotoxicity and by cell intoxication by misfolded and aggregated SOD1 protein conformers [21]. Excessive glutamate release and the disturbance of glutamate homeostasis are also associated with cerebral ischemia (stroke), traumatic brain injury, multiple sclerosis, epilepsy, migraine, and Alzheimer’s disease [21].

In summary, there is compelling evidence from the literature that acute and chronic oral exposure to high levels of glutamate or glutamate analogs can induce significant adverse effects on neural function. Reversible, persistent, and progressive motor and cognitive disorders are associated with glutamate-receptor-mediated excitotoxicity [22,23]. We therefore support the call for further studies to address the long-term safety of exposure to MSG.

### 3.2. Description of Cases Involving Lupin Seeds and Links with Alkaloids

In 2016, the Center of Rare Neuromuscular Disease (part of the Neurological Reference Center of Grenoble CHU Hospital in France, 38,000) examined the 56-year-old spouse of an ALS patient who reported an 18-month history of muscle fasciculation and cramping that varied in intensity from day to day. The neurological examination was normal, except for the presence of bilateral mydriasis. However, electromyography revealed massive fasciculation (5 per second) without any other expression of peripheral nerve hyperexcitability. Sleep was preserved. A life-habits questionnaire revealed that her muscle symptoms had begun over a one-year period during which she had substituted her usual vegan high-protein diet for one based on hand-made lupin flour. Cessation of the lupin diet for 4 months resulted in the complete resolution of her CFS.

We also encountered two patients who experienced a similar reversal of CFS linked to the excess consumption of lupin flour as a replacement of animal protein in a strict vegan diet. These patients were 41-year-old monozygotic twin brothers who, for ease of consumption and its high protein content, had consumed raw lupin flour in the context of a hiking trek. For five days, lupin had served as their only nutritional intake.

In all three CFS cases, lupin seed had been purchased via the Internet with packaging information that failed to state the lupin species, the origin of the seed, or methods for food preparation, notably detoxication. However, the seed of white lupin (*Lupinus albus*, L.) is widely available and has been consumed for many years in Western Europe by individuals wishing to substitute animal with plant proteins for cardiovascular disease prevention. Yellow lupin (*Lupinus luteus* L.) is used to feed livestock and poultry, although in Eastern Europe it is increasingly cultivated for its seed.

Motoneuron disease, with amyotrophy, fasciculation, diffuse hyperreflexia, and extensor plantar responses—all associated with the ingestion of toxic lupin seed—was described in French adults years ago by Agid and colleagues [24] and again more recently [25]. In the former, a 28-year-old female who had consumed lupin seed (3 g seed/month for 8 years) presented with dysarthria, hemi-dystonia, diffuse hyperreflexia, and extensor plantar responses that progressed over months to weakness accompanied by dysphagia and fasciculation of the limbs and tongue. The dystonic movements gradually disappeared, and, at this time, electromyography revealed polyphasic motor units mainly in the upper limbs. Twenty months after stopping the ingestion of lupin seed (probably *L. albus*), she was neurologically stable but exhibited pyramidal signs, weakness, and amyotrophy in all four extremities. Fasciculation was no longer present, the dysarthria had improved, and the dysphagia had resolved.

Lupin seed has sweet and bitter forms (https://www.feedipedia.org/node/23097, accessed on 10 July 2024), the latter resulting from a high content of quinolizidine alkaloids known for their potential anticholinergic toxicity. The acute symptoms of lupin intoxication, which are distinct from those of CFS, include weakness, teary eyes, mydriasis, and nausea, linked to anticholinergic syndrome; this may occur in adults who consume only two handfuls of lupin seeds [26], and children ingesting as few as ten lupin seeds may complain of blurry vision, headache, photophobia, mydriasis, and nausea [27]. No specific treatment of acute anticholinergic syndrome is needed, and symptoms usually resolve about 12 h after exposure. Mydriasis has also been mentioned as an acute symptom after lupin bean poisoning in Canada [28].

These anticholinergic symptoms show some diversity since they are dependent on the type and concentration of individual alkaloids present in lupin seeds, information that is unknown given a risk-assessment approach based on the total content of these toxic molecules. In fact, for lupin species, it is known that sweet and bitter varieties are present under different genotypes, but they lack any specific taxonomic traits that allow recognition of the different varieties [29]. Usually, white lupin (*Lupinus albus* L.) grown in Europe, narrow-leaved lupin (*Lupinus angustifolius* L.) grown in Australia, and pearl lupin (*Lupinus mutabilis* Sweet) grown in South America are used for human consumption. Lupin varieties are referred to as “bitter” when the total content of alkaloids is equal to or higher than 10 g/kg dry seed or as “sweet” when the content is lower or equal to 500 mg/kg dry seed. While advertised as a healthy high-protein (300 to 400 g/kg) dietary component, lupin seeds are deficient in methionine and lysine relative to FAO standards. The introduction of sweet lupin has widened the food use of lupin from the traditional use of debittered seeds as a snack food [30].

The bitter taste of some lupin varieties results from the presence of quinolizidine alkaloids, represented mainly by potentially toxic molecules such as anagyrine, sparteine, lupinine, and lupanine, among others [31,32,33]. Debittering is possible through different preparatory steps, including water soaking, cooking, and further soaking [33]. More than 35 years ago, an alert was issued to poison control centers and toxicologists describing the potential toxicity of “edible lupini” when debittering instructions are not followed [33]. A recent EFSA risk assessment [34] concluded that intoxication with lupin seed can occur, although infrequently, leading sometimes to a fatal outcome; in poisoning cases, the estimated total alkaloid intake is 10–50 mg/kg human body weight based “on human data from case reports”. Due to the limited data on occurrence and consumption, dietary exposure was calculated for some specific scenarios, but no full human health risk characterization was possible. The calculated margin of exposures (MOEs) may indicate a risk for some consumers, for example, when lupin seeds are consumed without a debittering step, such as debittered lupin seeds with a high quinolizidine alkaloid content or as a “lupin-based meat imitation” [34].

In France, in 1998, the Superior Council of Public Hygiene of France (CSHPF) proposed [35] an upper alkaloid limit of 200 ppm (200 mg/kg flour) in lupin flour. This seems to be a protective threshold since (i) the LD 50 for lupin alkaloids administered experimentally to young hens [36] is close to 1 g/kg pullet bw (pbw); (ii) the lethal dose of sparteine in humans corresponds to 30 mg/kg human bw (hbw) (i.e., 2.1 g of sparteine per 70 kg hbw); and (iii) a case report for lupanine poisoning resulted from 25 mg/kg hbw of the alkaloid [37]. The foregoing human fatalities would have ingested 5–10 kg of lupin flour, an amount greatly in excess of the proposed limit for human alkaloid exposure. Furthermore, the CSHPF alkaloid limit is broadly in accord with regulations in Australia, New Zealand, and the UK [38,39]. Note, however, that these regulations seek only to prevent acute lupin seed toxicity and may have unknown relevance to chronic lupin toxicity leading to CFS or frank, self-limiting motor neuron disease.

The French Agency for Food, Environmental and Occupational Health & Safety (ANSES) classifies lupin seed in the list of “Toxic plants if swallowed” [38]. The German Federal Institute for Risk Assessment (BfR) recommends that consumers “who do not have sufficient knowledge of the subject avoid the consumption of lupin seeds not debittered by the manufacturer, as there is no certainty that the recommended debittering procedures result in a sufficient reduction in the levels of health-damaging alkaloids [39]. “If lupin seeds or products made from them have a bitter taste, this can be an indicator for the presence of undesired, health-damaging lupin alkaloids. The bitter-tasting water from soaking the lupin seeds for debittering should not be consumed under any circumstances or used for the preparation of foods”. Indeed, an anticholinergic toxic syndrome was reported after ingestion of only 100 mL of debittering water [40], with a similar case in France [26]. It is suggested that children are more susceptible (five times more) than adults, and doses >10 mg/kg bw should be considered to be lethal in children [27,28]. In sum, children should be forbidden from consuming lupin seed, especially those of *L. albus* or *L. luteus* [34]. Furthermore, lupin seed is recognized as a significant human allergen by European Union food regulations (since 2007) and in other jurisdictions [41]. The common use of lupin seed, especially in bakery and confectionary products, also poses this additional potential adverse health effect [42].

## 4. Conclusions

For patients with muscle cramps and fasciculations (CFS), it is recognized here that CFS may be linked to a specific dietary factor or following a recent change in diet composition. In fact, the CFS cases presented here are considered to be secondary to the consumption of either umami-containing food or lupin seeds. The known biochemical components in both foods may well explain the occurrence of CFS in these patients. For this reason, we consider that consumers and clinicians should be aware of the health risks raised by such consumption. Regarding the use of monosodium glutamate (MSG) as a food additive, moderate use appears to be appropriate, especially by respecting its acceptable daily intake (ADI) of 30 mg/kg bw/day. Furthermore, recently it was shown that glutamate may well be the master neurotransmitter responsible for shaping the entire brain [43]. A recent new perspective was given for combating glutamate excitotoxicity by developing therapeutic bioconjugates [44]. Further research is also required to identify which lupin chemicals may have chronic neurotoxic potential. Additionally, special attention should be given to the preparation and use of lupin seeds as a food item given their potential for toxicity leading to poorly reversible effects on motor neuron function. The use of recent techniques [45] should allow the identification and dosage of quinolizidine alkaloids implicated in lupin toxicity. Descriptions of other cases with CFS are urgently needed to help better define the clinical spectrum that may be observed in such intoxications.

## Figures and Tables

**Table 1:** Reported cases of CFS and associated observations.

	Clinical Factors	Intake	Weight Intake	Time Interval between Intake and Symptoms	Duration of Symptoms after Intake Stopped	Cramp–Fasciculation-Associated Symptoms	Previous Susceptibility
Case 1	54, woman	umami	>200 g	20 min	3 days	Facial flush Hot flashes Migraine Anxiety Chest oppression	Yes, migraine with soya sauce
Case 2	48, woman	umami	~200 g	15–20 min	3 days	Facial flush Hot flashes Hypersalivation Vertigo Feeling of suffocation	Low, irregular migraine with Roquefort cheese
Case 3	36, woman	umami	~200 g	15–20 min	3 days	Facial flush Migraine Syncope/vertigo Anxiety	No
Case 4	56, woman	Lupin	10 g/day over 1 year	1 month	4 months	Mydriasis	No
Cases 5 and 6 (twins)	41, men	Lupin	200 g/day over 5 days	1 day	15 days	Mydriasis Nausea	No

## Data Availability

The datasets generated in this study are available upon reasonable request from the corresponding author.
